# Acute coronary syndrome in a patient with coexistent conduction abnormality

**DOI:** 10.3402/jchimp.v1i1.7016

**Published:** 2011-04-19

**Authors:** Marc Mugmon

**Affiliations:** Department of Medicine, Union Memorial Hospital, Baltimore, MD, USA

A 46-year-old man had a 1-week history of exertional chest tightness and dyspnea. His symptoms became especially prominent while shoveling snow and he presented to the emergency department. He was free of pain at the time of initial presentation and denied any pain at rest (Fig. 1). One hour after reaching the emergency room his pain recurred and he became hypotensive and a second ECG was obtained (Fig. 2).

**Fig. 1:**
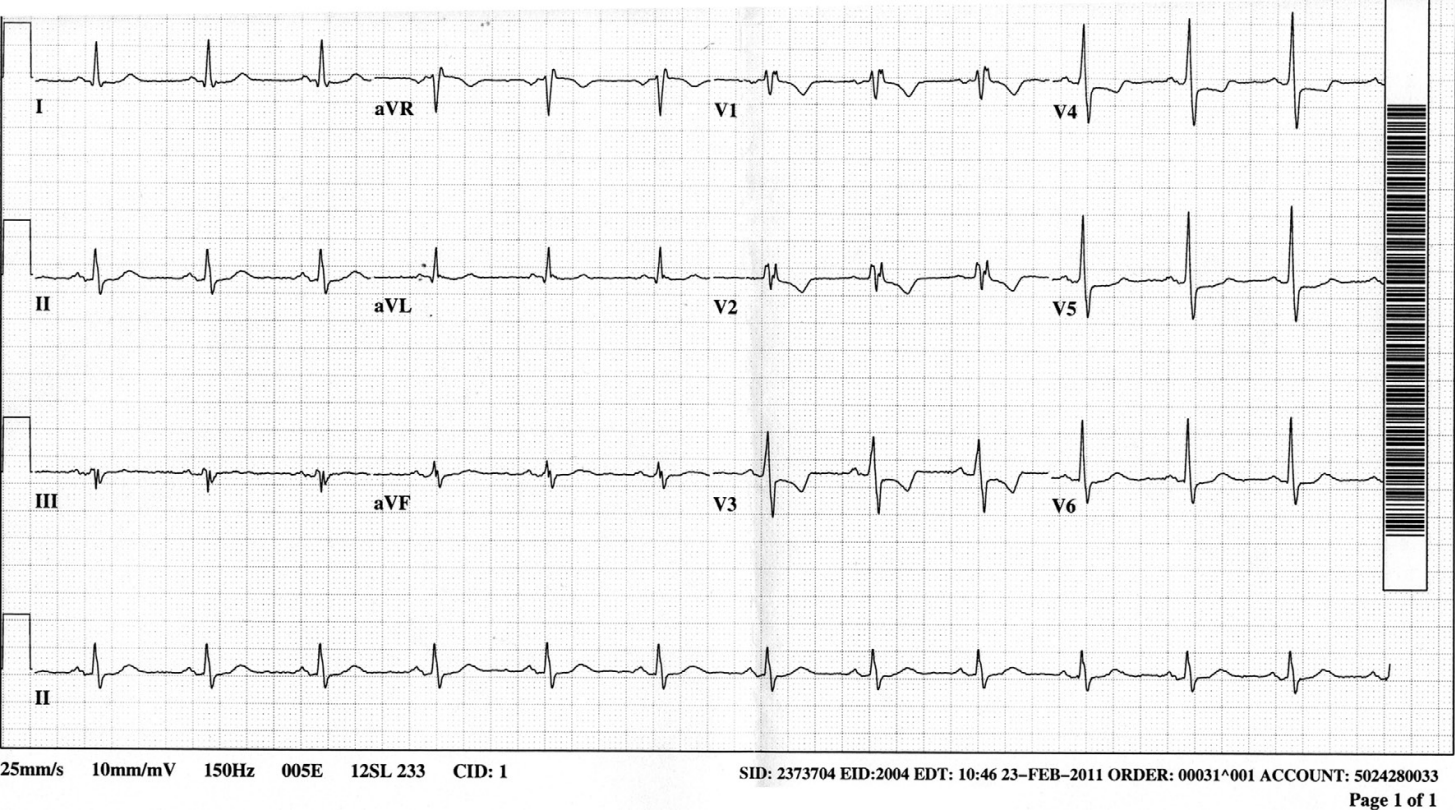
Electrocardiogram: ECG #1.

**Fig. 2:**
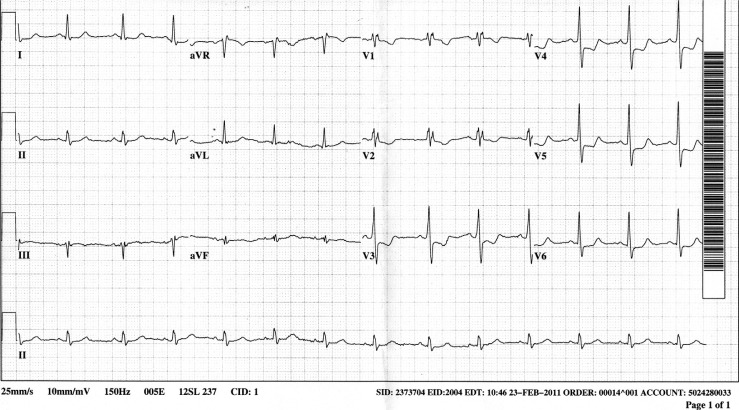
Electrocardiogram: ECG #2.

Past history was negative for hypertension, diabetes, and dyslipidemia, but he admitted to rare checkups. He had one presumed vasovagal episode years before, having passed out while laughing forcefully. There was a remote history of pyloric stenosis and abdominal hernia repair.

He was a lifelong non-smoker and denied significant alcohol consumption or any drug use.

He was on no medications.

Examination revealed a moderately overweight male (BMI 28).

BP 120/70 P 78 R 18

Physical examination was normal.

Cardiac biomarkers were normal.

ECG #1 (Fig. 1): Sinus rhythm with slightly increased QRS (112 msec)

Incomplete right bundle branch block

ST depression V3–V5 compatible with anterior ischemia

ECG #2 (Fig. 2): ST depression is more pronounced in V3–V6 with ST depression becoming downsloping in V3 and V4

Given aspirin, heparin, integrilin, and sent to cardiac catheterization lab

Cardiac catheterization revealed a 99% stenosis of the left anterior descending and total occlusion of the circumflex, with collaterals supplying the distal circumflex (Fig. 3). The right coronary artery was totally occluded, with bridging collaterals supplying the distal vessel (Fig. 4). Left ventriculography revealed normal systolic function and no wall motion abnormalities.

**Fig. 3:**
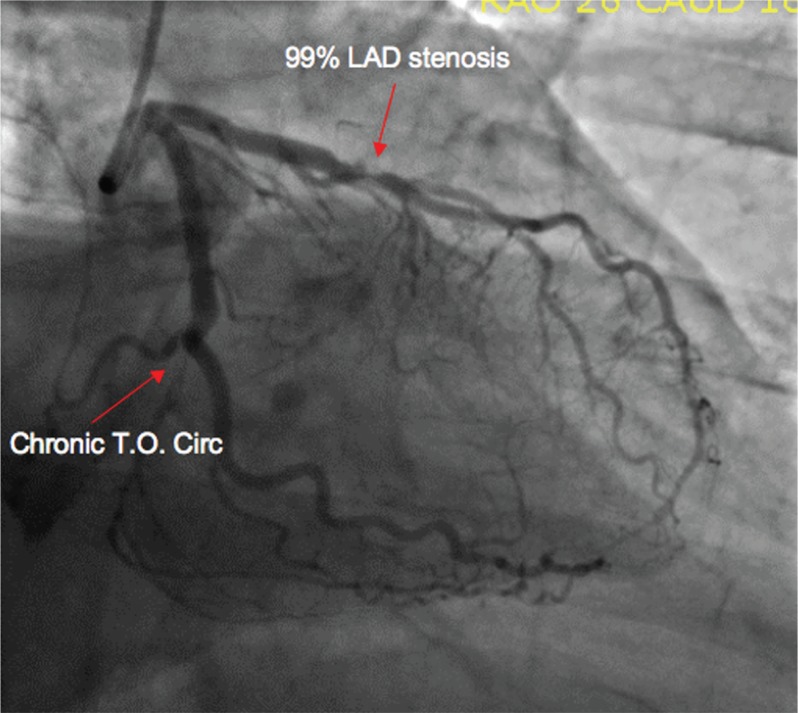
Angiographic images; 99% LAD stenosis and total circumflex occlusion with collaterals.

**Fig. 4:**
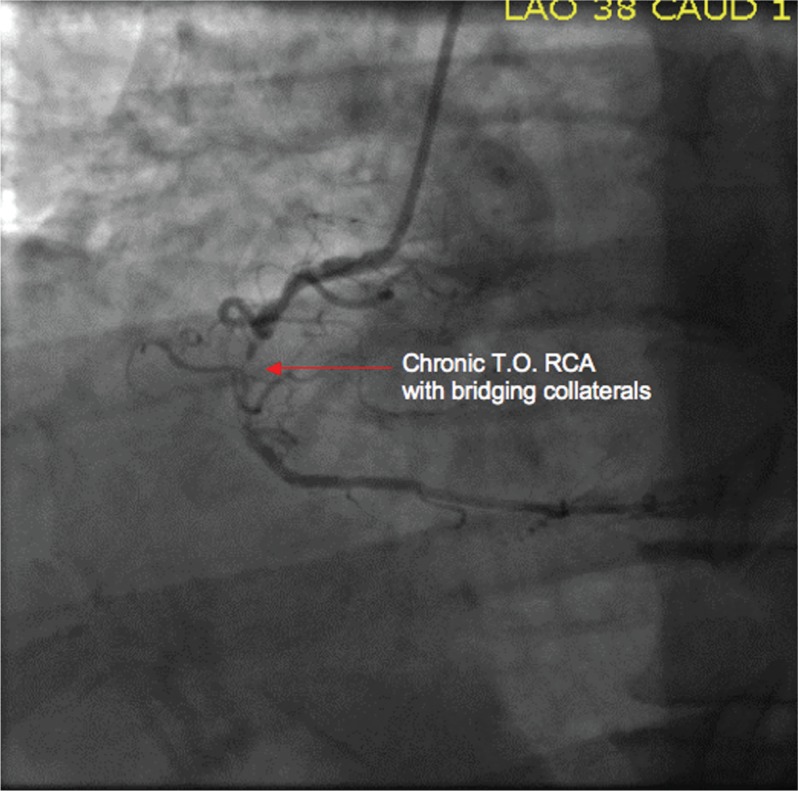
Chronic total occlusion RCA with bridging collaterals.

It was felt that the right and circumflex total occlusions were chronic and that the left anterior descending stenosis was more recent, explaining the sudden development of typical angina, followed by a rapidly progressive symptomatic course in the ER. A drug eluting stent was placed in this vessel and the patient became asymptomatic. He was discharged the next day on beta blockers, an ACE inhibitor, a statin, aspirin, and clopidogrel.

## Comments on the electrocardiogram

The most important findings in the initial tracing would be horizontal ST depression in V3 and V4, and T-wave inversion in lead V3, indicative of anterior ischemia, and very suggestive of a left anterior descending lesion.

An RSR’ (a so-called ‘little-big rabbit ears’) pattern is noted in leads V1 and V2. This pattern is normally seen with right bundle branch block (RBBB), in which the QRS duration is typically 0.12 secs or greater. If the same pattern is seen with a QRS duration of <0.12 secs, then incomplete right bundle branch block is present. Incomplete RBBB is present in up to 2.4% of normal individuals (1).

In both right and left bundle branch block, ST-T abnormalities are typically seen. They are characterized by the ST segments and T waves being in opposite direction to the main QRS deflection. In RBBB, the main QRS deflection is positive in V1, so the ST segment and T waves will be negative. In V6, the main portion of the QRS is negative, so conversely, the ST segment will be elevated and the T waves will be upright. These ST-changes are referred to as ‘secondary’ since they are due to the conduction abnormality.

In addition to being seen in complete BBB, secondary ST-T abnormalities occur in non-specific intraventricular conduction delays, pre-excitation, fascicular block (hemiblock), ventricular hypertrophy, ventricular ectopic beats, and ventricularly paced beats. All of these ST-T abnormalities are due to changes in ventricular activation.

In contradistinction to secondary ST-T abnormalities, primary ST-T changes are the result of processes that are *independent* of changes in ventricular activation. Possible causes include pharmacologic effects due to anti-arrhythmic agents, electrolyte disturbances, neurogenic effects (increased intracranial pressure due to hemorrhage), and myocardial ischemia or infarction.

This patient had primary ST-T abnormalities in V3 through V6 (due to demonstrable anterior ischemia). Secondary ST-T abnormalities in these leads would have been characterized by ST elevation. The ST-T abnormalities noted were opposite to those expected with the conduction abnormality and were due to ischemia.

The electrocardiogram the next day showed improvement in the anterior ST depression, but the T inversion in V2 persisted.

## Discussion

This case serves to illustrate how progressive coronary atherosclerosis can go undetected and can lead to minimal or no symptoms until the disease has progressed to a significant degree. It is likely that the right and circumflex lesions were present for years and that the development of collaterals prior to their becoming totally occluded allowed for the lack of symptoms. However, when the left anterior descending stenosis became critical, he developed typical angina, and presented for treatment prior to suffering what would have likely been a large infarction, considering the relatively large amount of myocardium supplied by this vessel.

Twenty to 25% of patients have potential coronary collateral channels in the absence of coronary stenosis, but in most they appear to be due to angiogenesis related to the development of ischemia (2).
